# Information Theory, Developmental Psychology, and the Baldwin Effect

**DOI:** 10.3389/fnbot.2018.00052

**Published:** 2018-09-04

**Authors:** Erick J. Chastain

**Affiliations:** Department of Ecology and Evolutionary Biology, University of Tennessee Knoxville, Knoxville, TN, United States

**Keywords:** evolutionary biology, developmental psychology, phenotypic plasticity, population genetics, information theory

## Abstract

As part of the extended evolutionary synthesis, there has recently been a new emphasis on the effects of biological development on genetic inheritance and variation. The exciting new directions taken by those in the community have by a pre-history filled with related ideas that were never given a rigorous foundation or combined coherently. Part of the historical background of the extended synthesis is the work of James Mark Baldwin on his so-called “Baldwin Effect.” Many variant re-interpretations of his work obscure the original meaning of the Baldwin Effect. This paper emphasizes a new approach to the Baldwin Effect, focusing on his work in developmental psychology and how that would impact evolution. We propose a novel population genetics model of the Baldwin Effect. First, the impact of a kind of learning process motivated by motor babbling, in the developmental psychology literature, on evolution; second, that Information-theoretic phenotype reshaping speeds up evolution compared to populations without this kind of learning. The basic idea behind the model is to allow the organism to apply abstraction to his initial phenotype to situate it within one of a few different classes of phenotypes in the local neighborhood of a fitness maximum. The reshaping of the phenotype space thereby allows the organism to reach a nearby fitness maximum. By so doing, valleys in the fitness landscape are leveled out, making a rugged fitness landscape into a set of mesas and plateaus with increasing height. Using this model we can show the first sizeable speed-up for the Baldwin Effect compared to ordinary population genetics. We also introduce an information-theoretic foundation for the Baldwin Effect, which may be of independent interest.

## 1. Introduction

Phenotypic plasticity (DeWitt and Scheiner, 2004), under its different aspects (learning, social and cultural innovation) has come into sharp focus recently as a possible source for genotypic change, with some going so far as calling this set of new ideas an extended evolutionary synthesis (Laland et al., 2015). Ordinarily in the classical evolutionary synthesis, genetic change arises from mutation, drift, or selection. The extended evolutionary synthesis seeks means by which phenotypic plasticity or phenotypic change more broadly speaking can affect genetic change, through processes like epigenetic inheritance and niche construction. Antecedents of the extended synthesis were pondered by James Mark Baldwin in his work on the Baldwin Effect. Baldwin showed that there could be a way that ordinary genetic change could occur in a direction controlled or “ratified” by phenotypic plasticity, but of a very specific form: abstraction of inputs, leading to a different kind of space in which phenotypes can be generated. Abstraction is the formation of more general concepts from specific examples, in contrast with learning. Learning is the process of associating stimuli with rewards, or the process of making good predictions (of new examples). Abstraction is the formation of novel representations that can broadly generalize more abstract features of stimuli in order to “understand” or simplify dealing with them in the future. In other words, abstraction is more like compression or unsupervised learning than ordinary learning (which is normally called supervised learning) (Bishop, 2006). Through the various forms of abstraction and developmental stages of organisms (as further elucidated by Piaget et al., 2013) the phenotype could change, often radically, leading to new representations and means for phenotypic variation. Finally, the very form of plasticity itself could evolve, and was heritable, according to Baldwin. But in a quirk of history, much of his original work, which was in developmental psychology, went unread. Instead, a new “Baldwin Effect” was broadcasted to Evolution researchers by Simpson (1953): a process by which non-heritable plastic traits would be replaced by specific fixed genetic factors (Scheiner, 2014). Others have followed up primarily along the same lines as Simpson, including Waddington's work on canalization (Waddington, 1953), and those who consider the effect of learning on genetic change (Hinton and Nowlan, 1987).

In this paper, the Baldwin Effect, as understood by Baldwin himself is analyzed with a close reading of his work in developmental psychology. We conclude that his intention was to show how new kinds of behaviors could develop via abstraction that would allow for very advanced information-processing by an organism with the rudiments of phenotypic plasticity. Secondarily, Baldwin showed how abstraction could impact the direction of genetic change. A crucial assumption he introduced is that organisms with phenotypic plasticity can try out motor actions and obtain reward signals in their juvenile state which are a good approximation to the fitness of the behavior (If the behavior is considered as a genetically-determined reflex).

Besides the problems of Baldwin Effect interpretation, there are questions about whether it even makes sense in ordinary population genetics. The usual interpretation of the Baldwin Effect, as elaborated by Hinton and Nowlan, is that by using learning algorithms one can reach higher fitness during one's lifetime, thus smoothing the fitness landscape for animals born with bad reflexes (and thus paving the way for those better reflexes to emerge quickly in the population). Recent work has exposed that the examples used by Hinton and Nowlan are easily and quickly solved by ordinary evolutionary dynamics (Santos et al., 2015). Therefore, the idea that the learning-based Baldwin Effect (as in Hinton's work) could somehow be more efficacious than ordinary genetic change via mutation, drift, and selection has yet to be shown. The impact of phenotypic plasticity on evolution, and its connection to the Baldwin effect, has been shown by Scheiner et al. (2017), Waddington (1953), and others. In this paper we focus specifically on two more novel influences of phenotypic plasticity on evolution: first, the impact of a kind of learning process motivated by motor babbling (Information-theoretic phenotype reshaping), in the developmental psychology literature, on evolution; second, that Information-theoretic phenotype reshaping speeds up evolution compared to populations without this kind of learning, all using ordinary population genetics. As shown by Szathmary, it was a known flaw in the older model of the influence by learning processes on speed of evolution that their speed-ups were not much faster than those associated with ordinary genetic drift.

A fitness landscape is the landscape which has its height defined by the fitness of the phenotype (as it varies over the space of all possible phenotypes). An example of an easy fitness landscape is a single hill, which can be climbed by natural selection. A medium-difficulty fitness landscape is a flat landscape with a peak (which requires some randomness but does not work against natural selection). A hard fitness landscape is a rugged landscape with many peaks (which requires randomness to work against natural selection). In this paper, the Baldwin Effect introduced is formally modeled and shown to significantly speed-up evolution in a rugged fitness landscape using ordinary evolutionary dynamics. Specifically, without the Baldwin Effect, the time for the most fit mutant to fix in the population slows down exponentially as fitness valleys get deeper. With the Baldwin Effect, there is no dependence of the time to fixation on the depth of the fitness valleys. The core insight is to show that the ability for organisms to undergo reward-based sensorimotor abstraction during their youth allows them to effectively flatten the hills in a rugged landscape. In human neonates, this process of play (as motor babbling Meltzoff and Moore, 1997) combined with intrinsic and extrinsic reward, allows them to reach novel representations for goals. The means by which abstraction can do this is by allowing the organism to “cluster” all possible phenotypes into those that are close to the same fitness maximum, and then “decode” or “assign” the initial phenotype to its cluster. Under the Baldwin Effect the reshaped rugged fitness landscape is a set of neighboring plateaus and mesas that are of increasing height. Effectively then a transitional mutant on the path to one with maximal fitness can arise as a neutral intermediate mutation rather than as a deleterious intermediate mutation. Of possible independent interest is a link established between the Baldwin Effect and a certain kind of information-processing that is nearly optimal for the Gaussian channel (in an Information-theoretic sense).

## 2. Mathematical background

The mathematical background necessary for the formal model is the models and tools of Information theory. To understand information theory, consider the following communication game. Alice is communicating to Bob with a continuous-valued signal. At each point in time, the signal is corrupted by Gaussian noise (with a mean of zero). Bob receives the noisy signal and must decipher with high accuracy Alice's message. In information theory this is called communication over a Gaussian channel. It seems difficult for Alice to communicate in such a way that Bob can reconstruct her message. But, intuitively, Alice could exploit redundancy, sending many different similar codewords for every single message sent. By exploiting redundancy Alice can thus hope that Bob, knowing this coding scheme, can find the codewords that correspond to the same message. The process Bob uses to find the corresponding codewords is called decoding.

In particular, Alice could send a numerical codeword of length *n* such that the average sum of squares (power) for each character of the codeword is bounded by ω. An example of a codebook along these lines is the random codebook, that is, one chosen at random. A Gaussian random codebook chooses for each message a random sequence of *n* values sampled from a zero-mean Gaussian distribution with variance ω−ϵ, with ϵ being positive and small (Cover and Thomas, 2012).

For decoding, Bob can take the corrupted codeword *z* and find the nearest codeword, that is the codeword *x* in the codebook that minimizes the Euclidean distance $D\left(x,z\right)=\overset{n}{\underset{i=1}{\sum }}{\left(x_{i}-z_{i}\right)}^{2}$. For the Gaussian random codebook, Bob must also declare an error if the power of the nearest codeword is not less than ω. Smith and Morowitz (2016) The coding and decoding algorithm just described is optimal for communication between Alice and Bob for the Gaussian channel, communicating messages accurately at the maximal rate possible.

## 3. Background

In this section is described a novel approach to interpret the Baldwin Effect within the framework of “Evolution of learning” using developmental psychology.

### 3.1. Interpretations of the baldwin effect

In this part of the paper we describe Baldwin's work and how it relates to the literature on the “Baldwin Effect.” Specifically, we define and interpret the terminology Baldwin used to describe the Baldwin Effect. We also connect Baldwin's work on developmental psychology and how the life history of the organism contributes to the Baldwin effect and evolution of the species. A connection is made between the learning-based Baldwin Effect literature and an approach inspired more by the kinds of learning processes highlighted by Baldwin in his developmental psychology work.

#### 3.1.1. Baldwin effect qua baldwin or impact of learning in a general sense acting genetically

Any ideas of Baldwin were conditioned by his time and place: our models should be based on modern ideas about genetics and development. As such we should be cautious to use his theories as-is. Rather we should try to use modern ideas about genetics and development to formulate our models, giving credit to Baldwin for having a germ of some of these ideas in a pre-genetic context.

Baldwin gives a very explicit example of how Organic selection in its general sense can influence the direction of natural selection and variation, respectively. Baldwin's example concerns the origin of grasping and how functional selection can influence it:

“We may imagine creatures, whose hands were used for holding on with the thumb and fingers on the same side of the object held, to have first discovered, under stress of circumstances and with variations which permitted the further adaptation, how to make intelligent use of the thumb for grasping opposite to the fingers, as we do now. Then let us suppose that this proved of such utility that all the young that did not do it were killed off; the next generation following would be intelligent or imitative enough to do it also. They would use the same coordinations intelligently or imitatively, prevent natural selection getting into operation, and so instinctive “thumb-grasping” might be waited for indefinitely by the species and then arise by accumulated variation” Baldwin (1902).

Inspired by the preceding, and adapted for modern genetics, what Baldwin describes is thus a two-stage process of

1. Generating novel behavioral phenotypes by combining or associating existing instincts based on abstraction.
2. If those instincts can be acted upon by functional selection to form novel phenotypes of high viability, then those organisms who can use functional selection (selection of high viability behaviors) starting with those instincts will be retained.

The first stage is thus a kind of phenotypic plasticity associating instincts, and the second is a learning process.

Then after mutation acts on this high-viability population one gets a new population which also is retained, starting from variants of the same instincts which lead to good phenotypes with functional selection. The new population could have new instincts that do better than the old instincts, and are closer to the phenotype that is produced by functional selection. If such a mutant arises in the population, it would have higher fitness and thus create a new population, after which the two-stage process continues. Baldwin points out that this process terminates with a population that has instincts that match what functional selection produced at step (1) of the first chain of two-stage processes that were kicked off by functional selection. We call this the Baldwin effect qua Baldwin, noting that it is not the same as what Baldwin described due to its being framed in the context of modern genetics. We wished to call this model the Baldwin effect qua Baldwin in order to honor that his writing on learning in developmental psychology was a major inspiration for its formulation.

If we compare this mechanism with the variety of Baldwin effects identified in the literature, we can say the following:

1. Niche construction (Griffiths, 2003)
  Niche construction takes a fitness landscape of genetic variations that exist in the population and reshapes it. A special case of this is “Social Heredity” in which cultural selection allows one to reshape the fitness landscape. In fact the process Baldwin describes, because it involves real novelty of the phenotypes, will reshape the whole space of phenotypes, and then reshape the fitness landscape. The emphasis in the mechanism above is focused more on learning and its effects on the fitness landscape.
2. Smoothing the fitness landscape with learning (Hinton and Nowlan, 1987)
  Closely related to the Niche Construction view, but with a stronger connection to the Baldwin Effect qua Baldwin is the work of Hinton & Nowlan. They showed that when one evolves in the space of bitstrings (strings of one's and zero's such as 11010), if one starts out with a bitstring with medium hamming distance from the optimal (medium error), then one will retain those because backpropagation learning can set the other positions accurately and thus increase viability. This then increases the fitness of the medium error types, leading to a smoothing of the fitness landscape. The Baldwin effect qua Baldwin differs in two respects from Hinton & Nowlan: the learning mechanism (as outlined in the previous section) and that in Baldwin's case the phenotype space itself is reshaped in such a way as to both generate the optimal phenotype and reshape the fitness landscape in this space to be much easier (either by being smoother or reducing mutational distance to the optimal type). To place the Baldwin effect qua Baldwin in the same setting, imagine one can come up with a different representation for bitstrings, one which is useful for the environment. Then the Baldwin Effect qua Baldwin will reshape the phenotype space from the original bitstring representation to the new representation, and in the new representation the fitness landscape is smooth or the number of mutations until one gets an optimal bitstring is smaller. We will describe a more advanced example of how the phenotype-reshaping version of the Baldwin Effect can speed up evolution in the Results section (section 4.1.1).
  The mechanism described above (Baldwin qua Baldwin) is a kind of model for the impact of learning on evolution, like Hinton and Nowlan's work, but it is focused on learning processes found in developmental psychology that are different than the backpropagation neural networks considered by them. Moreover, the genetics framework considered is modern genetics rather than genetic algorithms. We will discuss in the sequel the relation between Baldwin's developmental psychology work in motor learning as formalized here and more recent work by Meltzoff on motor babbling.
3. Genetic assimilation (Simpson, 1953; Waddington, 1953) In Genetic Assimilation (according to Livnat et al., 2014), there is some structure to the phenotype (modeled by say a boolean function *f*) that when one combines the various expression levels of some proteins with variables related to the environment one gets novel phenotypes. One tries to generate phenotypes that are varying levels of expressions for proteins which when presented with novel environmental inputs can generate novel responses (e.g., assignments to some inputs of the boolean function). Then the assignments to the inputs of *f* which lead to high viability generally are retained in the population. Here the mechanism of variation was to change the assignments to *f* that are genetically-controlled. But the mechanisms of change in phenotype is due to phenotypic plasticity, an environmentally-induced change which can be far from random. In the Baldwin Effect qua Baldwin, the variation itself is based on learning mechanisms, and actually reshapes the phenotype space, whereas in Genetic Assimilation, it is of a different kind of phenotypic plasticity. That is, the phenotype space in Genetic Assimilation doesn't get changed, say, from the space of bitstrings to the space of even or odd bitstrings. Whereas it does for the Baldwin Effect qua Baldwin.

#### 3.1.2. The impact of the baldwin effect qua baldwin in a general sense on variation and generation of novel phenotypes

Now for the origin of variations or novel phenotypes, Baldwin gives the example of a child learning how to write:

“Every child has to learn how to write. If he depended upon chance movements of his hands, he would never learn how to write. But on the other hand, he cannot write simply by willing to do so…. What he actually does is to use his hand in a great many possible ways as near as he can to the way required; and from these excessively produced movements, and after excessively varied and numerous trials, he gradually selects and fixes the slight successes made in the direction of correct writing” Baldwin (1902).

Note that in the above case, we have a decidedly non-random set of behavioral variations to choose from. In fact the child tries to approximate the best way to write and of these approximations, she chooses the best one. Then according to the mechanics of the previous section, one would imagine that the child would vary her movements more if she is closer to writing well. The picture given here by Baldwin accords with our model of the previous section.

In the next section we will present a formal approach to modeling the Baldwin Effect, both organic selection and functional selection. Then we will discuss how the Baldwin Effect can have impact on population genetics.

## 4. Results

This section describes a formal model based on the new interpretation of the Baldwin Effect described in the Background section.

### 4.1. Formal model of the baldwin effect

Consider that for organisms with phenotypic plasticity, the initial phenotype can change in response to environmental and other factors. In the context of Baldwin's observations, we introduce a two-stage model of phenotypic change which incorporates a life-history of rewards and a changed phenotype. Baldwin describes a process of phenotypic change which starts with the initial phenotype *P*_0_ containing the instincts alone. The organism then changes to phenotype *P*_*T*_ in response to rewards *R*_*T*_ received over the life history (of length *T*). The iterative dynamics of how *P*_0_ changes throughout each epoch in the life history is related to *P*_*T*_ as a difference equation is related to its solution. For simplicity, we omit a thorough treatment of iterative dynamics and instead focus on the final state *P*_*T*_ (though see Sandefur's book if interested Sandefur, 1993). In accord with the connection between reward and fitness assumed by Baldwin, the reward history *R*_*T*_ is at each epoch an approximation to the fitness of the corresponding phenotype. For instance, the last reward in *R*_*T*_ approximates the fitness of phenotype *P*_*T*−1_. Then the basic model of Baldwin's Organic selection is given by the equation

(1) $$\begin{array}{r} P_{T}=\Phi \left(P_{0},R_{T}\right) \end{array}$$

The approximation of the fitness by the rewards gives us the following relationship between the final phenotype and the history *F*_*T*_ of fitnesses:

(2) $$\begin{array}{r} P_{T}\approx \Phi \left(P_{0},F_{T}\right) \end{array}$$

With the introduction of fitness histories into the approximate dynamics, the penultimate phenotype *P*_*T*−1_ could have a fitness different than that of *P*_0_. If a number of initial phenotypes $P=\left(P_{0},P_{0}^{\prime },\ldots \right)$ converge to the same *P*_*T*_ under Φ for some *T*, then they form a natural class of Φ-equivalent phenotypes. If indeed a good number of phenotypes are Φ-equivalent, then the fitness landscape across all phenotypes ends up being over a new space of phenotypes. Since the phenotypes change under Φ at a rate rapid enough to affect the fitness of the organism (Note that rapid phenotypic change does not necessarily rule out significant later-life plasticity). The effective change in the space of phenotypes occasioned by the process inspired by Baldwin and formalized by Equation (2) could fundamentally change the way that populations evolve in the long-run. Reshaping of the phenotype space due to the Baldwin effect could therefore have a significant effect on the way that populations evolve. The impact on population genetics will be explored later. We would like to present a specific reshaping function Φ based on information theory.

#### 4.1.1. Information theory and phenotype reshaping

Now we turn to the reshaping function for Equation 2 that we wish to characterize. Let's call it the Info-theoretic reshaping function Φ_*i*_. For the communication game and all other background details about the information theory, refer to the Mathematical Background section. Assume that the *P*_0_ for the organism is defined by motor parameters for the initial instincts. The choice of motor parameters each correspond to ways of encoding an abstract class *c*∈*C* of functions that the animal may perform in its niche (with *C* being the set of all such classes). Posit that the organism is trying to communicate to its environment by its instincts what class *c* of function it would like to perform in the environment. Now consider that the environment during the organism's life history is trying to decode the class *c* of functions corresponding to the motor parameters, and that the appropriateness of the functions for the current environment determines fitness. Then assume that the motor parameters are subjected to some kind of additional Gaussian noise in their execution (for instance, noise due to wear and tear, heat noise). For the model that gives rise to the reshaping function Φ_*i*_, we merely assume there is close-to-optimal communication between the organism and the environment: with the organism communicating the class *c* of ecologically-relevant functions it wishes to perform near-optimally to the environment for the purpose of natural selection. With the organism achieving communication near-optimal both in communication rate and also accuracy. Then what kind of strategy should the organism use? The communication game over the Gaussian channel will give us the answer.

Recall that the proposed encoding function for the Gaussian channel was based on a random Gaussian codebook. Then for the near-optimal code, the *n* motor parameters θ encoding the class *c* are chosen at random, according to the Gaussian distribution (with zero-mean and variance ω−ϵ as in the communication game). Each of the randomly-chosen set of motor parameters θ would give a way of executing instinctually each function class *c*. Such a code is similar in spirit to models found in neural coding theory (Pouget et al., 2000), but we view motor parameters (and the neural populations that code for them) in this case as encoding more abstract functions than saccades in response to motion direction (as in Shadlen and Newsome, 2001). More abstract neural codes can be found for instance in the literature for value coding in LIP (Platt and Glimcher, 1999). The optimal decoding mechanism, according to information theory, is given by the nearest codeword Gaussian channel decoder used by Bob in the communication game (if we rule out decoded codewords that give rise to decoding errors by having power greater than ω). How can we model the decoding mechanism if the environment is trying to decode which function class a noisy set of executable motor parameters belongs to and its appropriateness for the sake of natural selection?

The decoding mechanism requires a suitable fitness function. Such a fitness function is defined according to the initial random choice of motor parameters θ^*c*^ encoding each class *c*. For a near-optimal decoder, the fitness of a set of motor parameters θ can be set inverse to the Euclidean distance between θ and θ^*c*^, *D*(θ, θ^*c*^), where θ^*c*^ is the nearest codeword. In words, the closer one is to the nearest codeword θ^*c*^, the higher one's fitness will be. We should also note that for every motor parameter setting, there will also be a corresponding abstract class *c* of function for the organism (and it will be closest in terms of Euclidean distance in the space of motor parameters θ^*c*^). A suitable fitness function for instincts is thus:

(3) $$\begin{array}{r} f\left(\theta \right)=\arg\underset{c}{\min}\exp\left(-D\left(\theta ,\theta^{c}\right)\right) \end{array}$$

which is a special kind of Gaussian fitness function, as introduced by Fisher (Fisher, 1999; Martin and Lenormand, 2006, 2015).

Given the fitness model, we can now define Φ_*i*_. Let Φ_*i*_ be a function which when given a sequence *R*_*T*_ of reward values that is increasing, provides an output phenotype which gives at least the same reward as the last value of *R*_*T*_. Any kind of dynamics that increases *R*_*T*_ with respect to the phenotype could do this (for example, multiplicative weight updates Arora et al., 2012, gradient ascent Boyd and Vandenberghe, 2004, etc.). Then by Equation 2 such a Φ_*i*_ when combined with a fitness function 3 will output a *P*_*T*_ such that *f*(*P*_*T*_)>*f*(*P*_*T*−1_), where *P*_*T*−1_ = Φ_*i*_(*P*_0_, *R*_*T*−1_) and *R*_*T*−1_ is the reward history found in *R*_*T*_ excluding its last element. Therefore, since the output phenotype ends up increasing the fitness each iteration, for some *T*, *P*_*T*_ will be a local maximum of the fitness. But this would mean for some *T*, $P_{T}=\theta^{c}$ corresponding to the original class of the *P*_0_ = θ, by the definition of the fitness (Equation 3).

For the kinds of dynamics that increase reward *R*_*T*_ over time, all of the phenotypes that lead to the same local maximum of the fitness are Φ-equivalent. Therefore the Φ_*i*_-equivalent phenotypes are those which are closest to the same θ^*c*^, according to the definition of the fitness function (Equation 3). So the Φ_*i*_-equivalent phenotypes are all parametrized by θ^*c*^, and thus we denote them with $P_{\theta^{c}}$.

Before proposing our model specifically, we would like to present some background information about play and its role in child development. The primary phenomenon we will introduce is body babbling. Body babbling was introduced by Meltzoff and Moore (1997) to account for the process by which babies do movements in a free and non-directed way in order to develop deliberate reaching at the age of 8 months. As defined by Meltzoff and Moore (1997),

“ In body babbling, infants move their limbs and facial organs in repetitive body play analogous to vocal babbling. In the more familiar notion of vocal babbling the muscle movements are mapped to the resulting auditory consequence; infants are learning this articulatory–auditory relation. Our notion of body babbling works in the same way, a principal difference being that the process can begin *in utero*. What is acquired through body babbling is a mapping between movements and the organ-relation end states that are attained.”

In particular, we propose the following model inspired by Baldwin's account of motor babbling (Meltzoff and Moore, 1997) (which he calls body babbling) during play.

We propose a Φ-approximate reshaping function based on the Multiplicative Weight Updates Algorithm (MWUA) (Arora et al., 2012). MWUA selects one of *k* different experts, choosing experts with higher probability when their advice leads to higher reward. The reward for an expert *i* at time *t* is its reward $r_{i}^{\left(t\right)}$. Note that the loss $r_{i}^{\left(t\right)}$ is a function that varies for different applications of MWUA, and in the case of the application in our paper is specified by Equation (5). The probability distribution over experts $p_{i}^{\left(t\right)}$ at time *t*+1 for MWUA is given by:

(4) $$\begin{array}{r} p_{i}^{\left(t+1\right)}=p_{i}^{\left(t\right)}\frac{1+\eta r_{i}^{\left(t\right)}}{\sum_{j}p_{j}^{\left(t\right)}\left(1+\eta r_{j}^{\left(t\right)}\right)} \end{array}$$

with η>0 being the learning rate. When η is small, experts are chosen more based on long-term increases in reward, and when it is large they are chosen based on immediate reward.

The particular model for a Φ_*i*_-approximate reshaping function proposed takes the reward functions $r_{i}^{\left(t\right)}$ for MWUA to be the reward expected from exploratory play during some motor or navigation task. Each “expert” is a motor behavior. Now the reward function is assumed to be as follows:

(5) $$\begin{array}{r} r_{i}^{\left(t\right)}=-\arg\underset{c}{\min}\exp{\left(i-c\right)}^{2} \end{array}$$

where *c* is a particular target motor behavior that is the “goal” for the exploratory play, as defined by the nearest local maximum to the instinctual initial motor behavior strategy (at time *t* = 0, the motor behavior with highest probability $p_{i}^{\left(t+1\right)}$) on the corresponding fitness function Equation (3). It is a property of the MWUA that it converges in linear time *T* to the expert *i* that maximizes the cumulative reward $\overset{T}{\underset{t=1}{\sum }}r_{i}^{\left(t\right)}$. Arora et al. (2012) So therefore our model of motor play is a Φ-approximate reshaping function, since the motor behavior $P_{T^{*}}$ that the MWUA model converges to is the same as the local maximum, and thus satisfies the criteria for an approximate Φ_*i*_ information-theoretic reshaping function.

There have been many useful models of body babbling that have been proposed as of late in the robotics literature Lee (2011), making new advances in solving the inverse problems involved in motor planning (Rolf et al., 2010) and representation issues in sensorimotor representations (Law et al., 2013). We view our model as a simplified form of model for body babbling that allows us to ask what kind of impact it has on evolution of animals that engage in it and robots that use genetic algorithms combined with body babbling.

Motor and object play (Smith, 2010) are relevant to us, since they are a set of open-ended, non-goal-directed actions, like what would be found in the MWUA model with medium or low values of the temperature (for motor behaviors having to do with arm movement or object manipulation). Also, the MWUA model assumes there are internal reward signals associated with different motor behaviors, and that the ones which are closer to goal-directed are internally rewarded this way. So too does Lee (2011) emphasize the importance of internal rewards and intrinsic motivation as a way to model play, with the latter originally introduced by Furth (1969).

Sensorimotor development happens in stages, in order to set progressively harder learning problems to solve. Past algorithmic approaches have used these stage-wise sensorimotor constraints to model infant development during play, and in fact learned using appropriate constraints (Law et al., 2013). We too have considered the same with only one stage of constraints (modeled by the nearest goal-directed action) modeled by one round of phenotype-reshaping, but for multiple stages, there could be multiple rounds of phenotype-reshaping for complex goals.

Now we turn to the impact of phenotype-reshaping using Φ_*i*_ on the rate of evolution.

#### 4.1.2. Impact on population genetics

**Figure 1:**
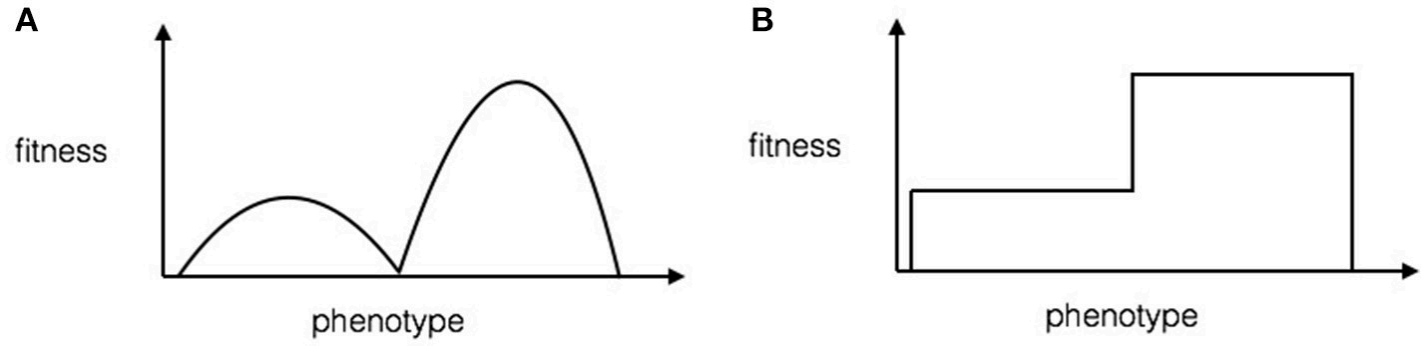
A toy illustration for the effect of phenotype reshaping on fitness landscapes. **(A)** Fitness landscape without phenotype reshaping according to the model. The *x*-axis is the continuous-valued phenotype, and the *y*-axis is the fitness. **(B)** Fitness landscape with phenotype reshaping. The *x*-axis is the continuous-valued phenotype, and the *y*-axis is the fitness. Note that phenotype reshaping flattens the hills in the rugged landscape.

As reviewed in the Introduction, there is currently no mathematical proof that something like the learning-based Baldwin Effect (as in Hinton's work) can speed up evolution in non-trivial ways. In this section we show that under the phenotype-reshaping account of the Baldwin Effect we can prove that there is a significant speed-up in the evolution of a complex trait. Phenotype reshaping can speed up evolution by effectively removing the stochastic element of crossing fitness valleys in a rugged fitness landscape. The mechanism for this is to reshape the fitness valley so that it increases in fitness to that of the local maximum, effectively just leaving a series of plateaus of ever-increasing fitness (see Figure 1).

Normally, in the evolution of complex traits, there are three regimes: the fast near-deterministic regime of evolving a trait with greater fitness after a single mutation, the intermediate regime of evolving a trait after a few steps of neutral or near-neutral evolution, and the slow stochastic regime of evolving a beneficial trait that requires a large decrease in fitness as an intermediate step to achieving the larger fitness (Weissman et al., 2009). For small population sizes, the first is called a beneficial mutant, the second is called sequential neutral fixation and the latter (for a *k*-allele gene) is called a beneficial *k*-mutant that results from sequential deleterious fixation. Sequential fixation regimes are so named because they involve small populations that have to sequentially fix intermediate mutations along the way to the final, beneficial, mutation.

If the difference in fitness between the original phenotype and the beneficial mutation is *s*, then the beneficial mutant arises in γ/*s* time (on average), where γ = 0.577… is Euler's constant (Desai and Fisher, 2007). Assume for *k* = 2 that the population size *N* is small, a single mutation will reduce the fitness by a large amount δ, and the double mutant will increase the fitness by a factor of *s* (which is called the deleterious sequential fixation regime). Then for a beneficial double-mutant with all mutation rates equal to μ to arise it takes approximately $\frac{1}{N\mu \rho_{1}}$ time (on average), (where $\rho_{1}=\frac{e^{\delta }-1}{e^{N\delta }-1}$). One can also look at a situation in which there are almost-neutral (δ < 1/*N*) or neutral intermediate phenotypes with a beneficial complex trait resulting from their combination, which is called a sequential neutral fixation process. For this setting with *k* = 2 necessary mutations the time for a beneficial mutant to arise is approximately $\frac{1}{\mu }$ on average. Such a regime is called neutral sequential fixation (Weissman et al., 2009). According to the work of Weissman, the deleterious sequential fixation regime takes more time to produce a beneficial double-mutant than the neutral sequential fixation regime, due to negative selection on the intermediate mutant.

Phenotype reshaping puts all steps of complex trait evolution into the beneficial mutant or the sequential neutral fixation regimes, with each step for the evolution of a trait reducing to a single mutation or a set of neutral steps to a beneficial mutant (traveling from the neighborhood of the local maximum to another adjacent neighborhood in one step). Effectively the evolution within a neighborhood of the local maximum is neutral, and so one individual on the boundary can arise and then cross over without any delay. In contrast, more time is required for the evolution of a *k*-beneficial mutant in the sequential deleterious regime. (Due to negative selection of the intermediates as they arise sequentially.) The next section describes a worked-out analysis of the speed-up for a simple example fitness landscape.

#### 4.1.3. Phenotype reshaping's impact on the speed of evolution for a simple example

The model of the last section, information-theoretic phenotype reshaping (using Φ_*i*_), is applied in this section as a means by which one can speed up evolution on a specific example fitness landscape. We show that if information-theoretic phenotype reshaping, as introduced in the last section, is used during the lifetime of those in the population, they can effectively flatten bumps on fitness landscapes and thus avoid fitness valleys (which slow down evolution). Thereby using info-theoretic phenotype reshaping functions Φ_*i*_ one can show on a simple example that evolution speeds up when one has this kind of information-theoretic phenotype reshaping.

The following example is not meant to model the genetic basis of behavioral traits or learning in general. It is a simple model which serves as a proof of concept that for behavioral traits that involve rugged fitness landscapes and Information-theoretic phenotype reshaping one can find a simple genetic mechanism based on population genetics that speeds up evolution considerably.

Consider a fitness landscape in which each phenotype is a bit-string of length *k*. For example, for *k* = 5 a phenotype would be 01101. All but one phenotype *x* will be either an optimal type, with fitness $\underset{i}{\sum }\left(x_{i}\right)$, or a phenotype which is suboptimal, with fitness $\frac{\left(1-c\right)\left(1+\underset{i}{\sum }\left(x_{i}\right)\right)}{e}$ where *c* is a positive constant. This is a special case of the fitness function proposed in the last section (Equation 3) with a Hamming distance function rather than a Euclidean distance. Despite the use of Hamming distance, the fitness function would behave similarly without loss of generality to a fitness function using Euclidean distance. The optimal phenotypes will be the bit-strings that correspond to even numbers, and the suboptimal ones will correspond to odd numbers (formally, if the number of 1's is even, then the bit-string is even, and likewise for an odd number of 1's and odd numbers).

**Figure 2:**
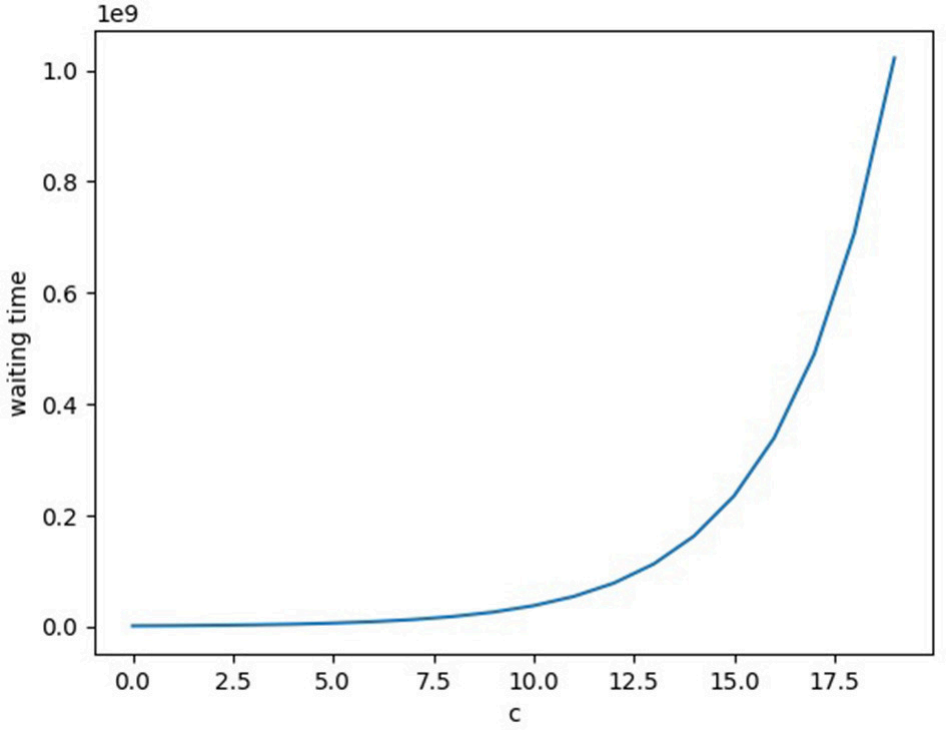
The waiting time for a double-mutant to arise (in the sequential deleterious fixation regime) for the simple example fitness landscape described in the main text. The *x*-axis is a parameter *c* that increases the depth of the fitness-valley, and the *y*-axis is the average expected waiting time.

For the analysis, rather than using the fitness as-is, the relative fitness is used. (Which re-normalizes the fitness of the optimal phenotypes to 1 and thus divides the suboptimal phenotype fitness by the original fitness of the optimal phenotype.) Then after phenotype reshaping as described above with Φ_*i*_, it will take at least *k*/μ time (on average), as evolution will happen in the sequential neutral fixation regime for double-mutants, and only *k* of those steps would be necessary. The reason the beneficial mutants are 2-away is due to the hamming distance between any even and odd bitstring being exactly one, so the distance between two optimal phenotypes is two. (A bit flip away from the optimal phenotype and a bit flip from the suboptimal phenotype to the next optimal one.) Along the same lines as the above argument, one can see that for the deleterious sequential fixation regime the double-mutant will take at least $\frac{k}{N\mu \rho_{1}}$ time (on average) to arise, where *N* is the population size and ρ_1_ is as above (with $\delta =1-\frac{1-c}{e}$). The final expression for the waiting time without phenotype reshaping simplifies to $\frac{1}{2\mu }\left(1+\exp\left[\frac{1}{e}\left(c+e-1\right)\right]\right)$. Now the waiting time is exponentially increasing in *c*. Comparing the two, the phenotype reshaping has as its rate of evolution something independent from the depth δ of the fitness valley, whereas the ordinary evolutionary rate slows down exponentially as a function of δ, see Figure 2. Analytically, the difference between the two regimes' waiting times is $\frac{k}{2\mu }\left(1+\exp\left[\frac{1}{e}\left(c+e-1\right)\right]\right)$, and thus grows exponentially in *c*. So the phenotype reshaping has a large impact on increasing the speed of evolution for this simple example, and for these biologically realistic parameter settings the effect grows linearly in the dimensionality *k* of the phenotype space.

## 5. Discussion

The Baldwin Effect is probably one of the most multifarious topics in Evolution. In this paper the many divergent interpretations were reviewed and a novel one was proposed. It seems that much of the literature has under-estimated the Baldwin Effect, due to the over-emphasis on genetics and the under-emphasis on developmental psychology. The role of abstraction over phenotypes in particular has been left out of most accounts of Baldwin's work on genetics.

Moreover, a phenotypic plasticity and abstraction-based account of the Baldwin Effect has other benefits. Notably, using some insights from Baldwin for the impact of play on evolution we were able to show the first Baldwin Effect-induced dramatic speedup of evolution on a fitness landscape using ordinary population genetics. The result is a notable improvement over prior work, which was based on neural networks theory and genetic algorithms and did not show dramatic improvement over ordinary evolution.

The most salient aspect of the Baldwin Effect we did not touch on in great detail was its emphasis on consciousness and its impact on genetics. We attempted to interpret what Baldwin meant by these effects by emphasizing the role of abstraction. There is nonetheless a gap between abstraction and what Baldwin seems to mean by consciousness, since he says that reason “ratifies” the moves proposed by genetics, and attention also has a role. But most of all Baldwin emphasizes the role of conscious experience in first-person control of innovation and behavior, and we have not explored those in any detail. It would be fascinating to explore the role of conscious experience in the Baldwin Effect in more detail.

Abstraction-based accounts of reason though are very old indeed, and go back all the way to Aristotle (2015) and Aquinas (1947). In addition, there is a rich tradition of abstraction-based structures informing the origin of biological innovations in the medieval literature on the scala naturae (Lovejoy, 2011). The scala naturae posits a set of major transitions based on new abstractions introduced at ever-higher “rungs” of the ladder. (With each rung being a kind of organism, for instance animals with sentience or plants with the ability to grow and self-repair.) Along these lines, recent work has tried to find rapprochement between Piaget's stages of child development and new formulations of the Baldwin Effect (Burman, 2013).

James Mark Baldwin was a pioneer in Evolution, but his primary advance was to explore the effect of developmental psychology on biological theory and function. Perhaps the most important work yet to be done is to bring more recent theory from developmental psychology (such as Gopnik's work on Bayesian theory; Gopnik et al., 2004; Gopnik and Tenenbaum, 2007) to bear on genetics. Such an update of the Baldwin Effect would be an interesting and natural direction left open by this work.

## Author contributions

EC ran experiments, did analysis, and wrote the manuscript.

### Conflict of interest statement

The author declares that the research was conducted in the absence of any commercial or financial relationships that could be construed as a potential conflict of interest.
